# Stress Granules Contain Rbfox2 with Cell Cycle-related mRNAs

**DOI:** 10.1038/s41598-017-11651-w

**Published:** 2017-09-11

**Authors:** Chungoo Park, Sunkyung Choi, Yong-Eun Kim, Siyeo Lee, Su-Hyung Park, Robert S. Adelstein, Sachiyo Kawamoto, Kee K. Kim

**Affiliations:** 1 School of Biological Sciences and Technology, Chonnam National University, Gwangju, 61186 Republic of Korea; 2 Department of Biochemistry, Chungnam National University, Daejeon, 34134 Republic of Korea; 3 Graduate School of Medical Science and Engineering, KAIST, Daejeon, 34141 Republic of Korea; 4 Laboratory of Molecular Cardiology, National Heart, Lung, and Blood Institute, National Institutes of Health, Bethesda, MD 20892 USA

## Abstract

Rbfox RNA-binding proteins play important roles in the regulation of alternative pre-mRNA splicing, but their role in other gene regulatory mechanisms is not well understood. Here, we show that Rbfox2 is a novel constituent of cytoplasmic stress granules, the translational silencing machinery assembled in response to cellular stress. We also show that the RNA binding activity of the Rbfox family protein is crucial for its localization into stress granules. To investigate the role of Rbfox2 in stress granules we used RNA-immunoprecipitation sequencing to identify cytoplasmic transcriptome-wide targets of Rbfox2. We report that a subset of cell cycle-related genes including retinoblastoma 1 is the target of Rbfox2 in cytoplasmic stress granules, and Rbfox2 regulates the retinoblastoma 1 mRNA and protein expression levels during and following stress exposure. Our study proposes a novel function for Rbfox2 in cytoplasmic stress granules.

## Introduction

Cells show significant gene-regulatory flexibility that allows them to adjust to external conditions. The ability to control gene expression plays an important role in the adaptation of cells to changes in the environment. Regulation of gene expression at the post-transcriptional level, including the control of pre-mRNA splicing, RNA transport, RNA stability and translation provides a rapid and efficient process by which the proteome of a cell can adapt to environmental changes^1^. RNA-binding proteins (RBPs) control various steps of nuclear and cytoplasmic RNA processing at the post-transcriptional level^2^. RBP-targeting to a RNA transcript affects RNA fate through various mechanisms that enable specific cellular functions to be established and provides a mechanism for post-transcriptional gene regulation during many biological processes. As RBPs have been implicated at various RNA processing steps and RNA metabolic events, it is therefore important to consider the cellular context in order to understand how RBPs exert effects on target RNA.

The RNA-binding Rbfox family is highly conserved with established roles in alternative pre-mRNA splicing regulation^3, 4^. The Rbfox family in vertebrates consists of three genes: Rbfox1, Rbfox2, and Rbfox3. Rbfox1 is specifically expressed in heart, skeletal muscle and neuronal tissues, whereas Rbfox2 is widely expressed in many tissues throughout life, from the early embryonic stage to the fully-grown adult stage^5, 6^. Rbfox3 expression is only detectable in postmitotic neurons of neural tissues^7^. All Rbfox family proteins contain a single RNA-recognition motif (RRM) in the middle of the molecule that binds to the hexanucleotide (U)GCAUG with a high affinity *in vitro*, and binding of Rbfox proteins to this element has also been well demonstrated in intact cells and tissues^3, 5, 8–14^. Numerous reports involving depletion of Rbfox in cultured cells and animal models have shown that the regulation of tissue-specific-splicing by Rbfox is critical to a number of biological processes^13, 15–19^. Recent reports show that the role of Rbfox is not limited to regulating alternative splicing in the nucleus but also reveal other nuclear and cytoplasmic functions for Rbfox proteins^20–23^. The enrichment of Rbfox binding sites in the 3′ UTR of many mRNAs was confirmed at a genome-wide level, and it has been shown that its targeting to the 3′ UTR stabilizes the target mRNA^12, 20, 24^. However, the precise function of Rbfox in the cytoplasm is largely unknown. Moreover recent studies have revealed other RNA binding sequences different from the consensus (U)GCAUG sequence *in vitro* and in intact cells^14, 22^.

The interaction between RBPs and RNA granules regulates the stability and translational efficiency of mRNAs and plays an important role in the regulation of protein expression in response to external stimuli^25, 26^. Stress granules (SGs) are active assemblies of messenger ribonucleoprotein particles (mRNPs), which are formed in the cytoplasm of cells under many different types of environmental stress^27^. SGs are non-membranous structures formed through stress-induced phosphorylation of the eukaryotic initiation factor (eIF2). Phosphorylation of eIF2 results in impaired translational initiation and a consequent decrease in protein synthesis, and at the same time mRNAs selectively incorporated into SGs show increased stability^26, 28–30^. SG assembly requires a subset of mRNA-associated RBPs such as T-cell internal antigen 1 (TIA1) and Ras-GTPase-activating protein SH3-domain-binding protein 1 (G3BP1). Moreover, multiple post-translational modifications of various SG components affect SG assembly^31^. Recent studies show that several components of SGs exist in the cytoplasmic granules of neuronal cells in the brains of patients with various neurodegenerative diseases including Alzheimer′s disease, Creutzfeldt-Jakob disease, and Huntington’s chorea, suggesting that the proper formation of SGs is important for neurological function^32, 33^. While the precise mechanisms by which SGs influence various biological processes including human diseases are largely unknown, it is clear that the post-transcriptional mechanisms mediated by SG-associated proteins and RNAs play a major role in enabling cells to adapt to external stimuli.

In this study, we investigate the cytoplasmic function of Rbfox2 under stress conditions. Unexpectedly, we found that Rbfox2 is a component of SGs and thus set out to identify the endogenous target of Rbfox2 in the SGs at the transcriptome scale, using RNA-immunoprecipitation sequencing (RIP-seq). This analysis revealed that cell cycle-related mRNAs were over-represented as cytoplasmic Rbfox2 targets under cell stress.

## Results

### Rbfox2 is a component of SGs

**Figure 1:**
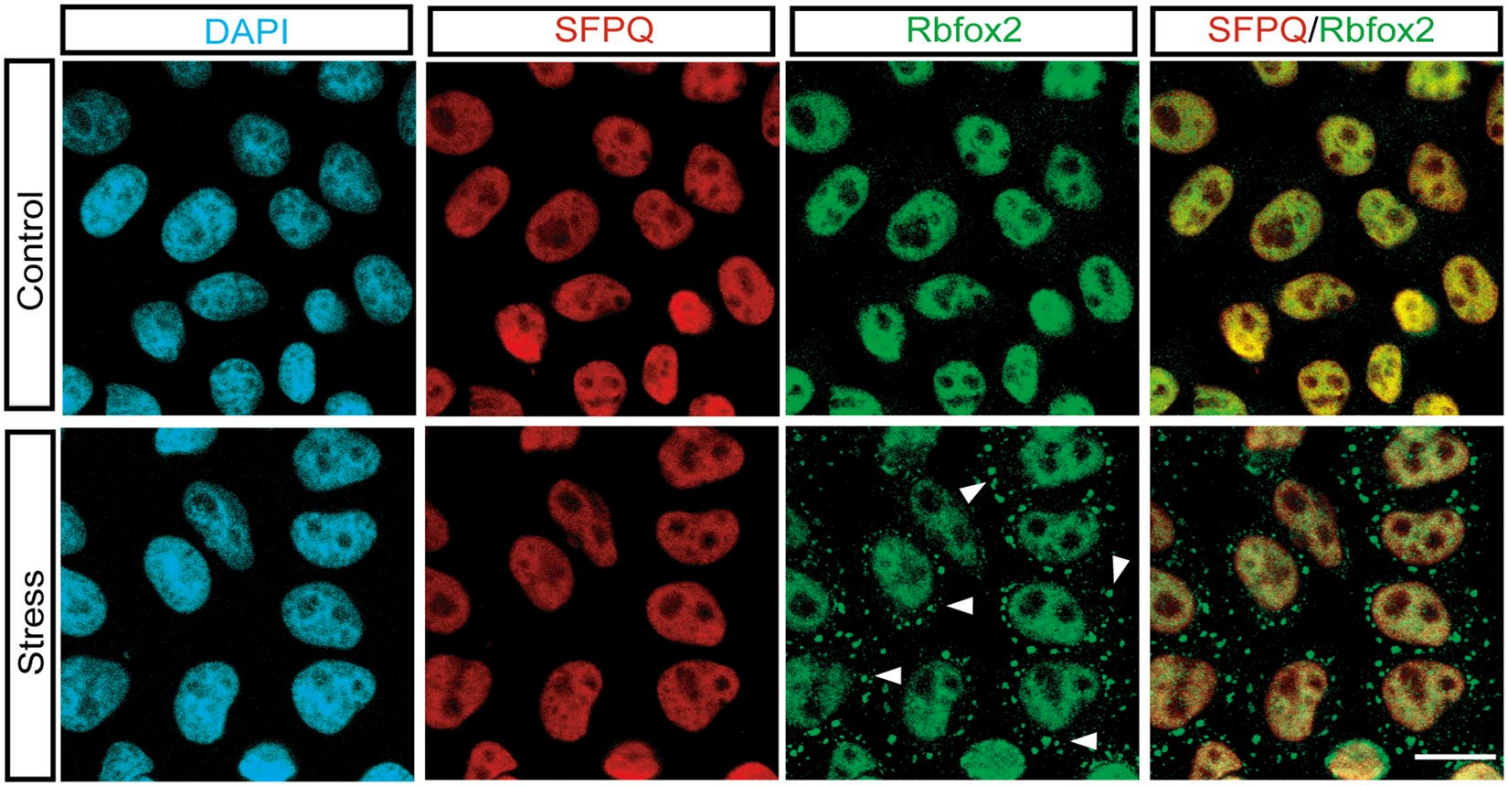
Sodium arsenite treatment induces cytoplasmic Rbfox2 granules. Immunofluorescence images of SFPQ (red) and Rbfox2 (green) proteins were visualized in untreated (Control) and sodium arsenite-treated (500 μM; 40 min) HeLa cells (Stress). DAPI stains nuclei. Arrowheads indicate cytoplasmic Rbfox2 granules. Scale bar, 20 μm.

**Figure 2:**
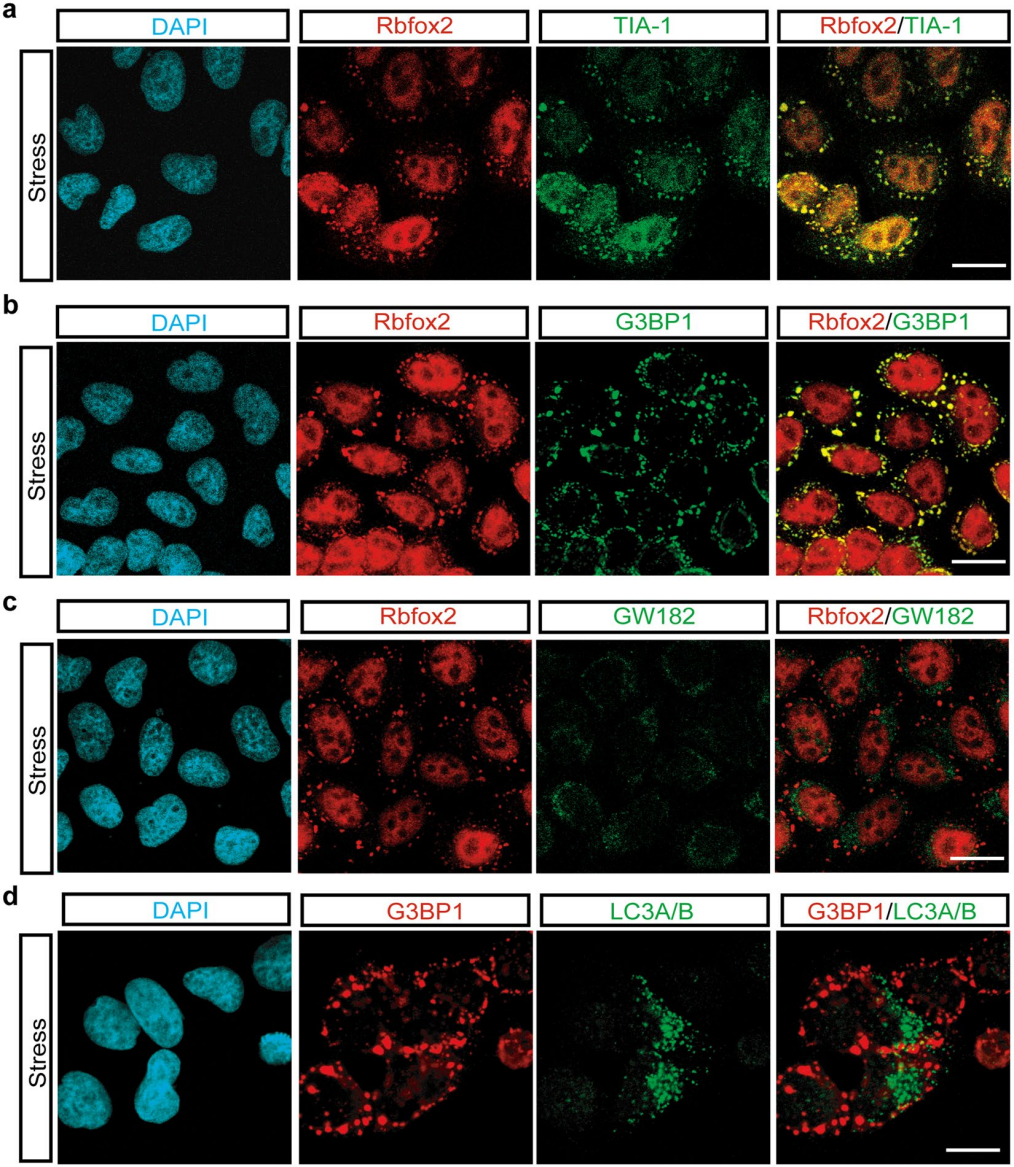
Recruitment of Rbfox2 protein into SGs by sodium arsenite treatment. (**a**) Immunofluorescence images of Rbfox2 (red) and TIA-1 (green) proteins were visualized in sodium arsenite-treated (500 μM; 40 min) HeLa cells (Stress). (**b**) Immunofluorescence images of Rbfox2 (red) and SG marker G3BP1 (green) proteins in sodium arsenite-treated HeLa cells. (**c**) Immunofluorescence images of Rbfox2 (red) and P-body marker GW182 (green) proteins were visualized in sodium arsenite-treated (500 μM; 40 min) HeLa cells (Stress). (**d**) Immunofluorescence images of G3BP1 (red) and autophagy marker LC3A/B (green) proteins were visualized in sodium arsenite-treated (500 μM; 40 min) HeLa cells (Stress). DAPI stains nuclei. Scale bar, 20 μm.

**Figure 3:**
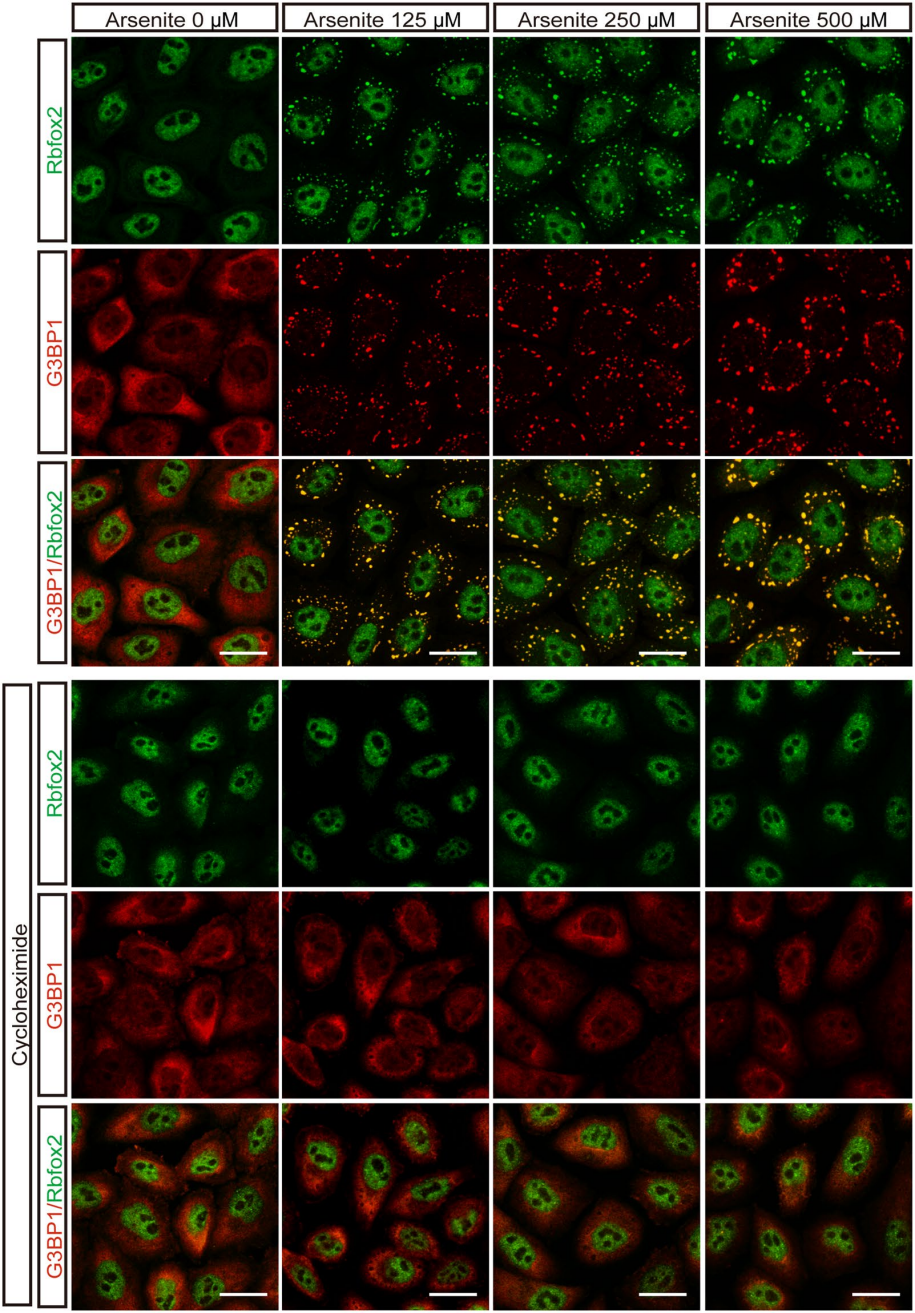
Inhibition of Rbfox2-containing SG formation by cycloheximide. HeLa cells were co-treated with indicated arsenite concentrations and 50 μg/ml concentration of cycloheximide for 40 min. Immunofluorescence images of Rbfox2 (green) and G3BP1 (red) proteins were visualized. Scale bar, 20 μm.

Rbfox2 is predominantly localized in the nucleus, as seen in human cervical carcinoma HeLa cells (Fig. 1). To investigate the role of Rbfox2 under stress conditions, we examined the subcellular localization of Rbfox2 following treatment with arsenite, a well-known oxidative stress inducer that has been shown to induce the formation of cytoplasmic SGs in a variety of different cell types^34^. Although nuclear localization of the splicing factor prolineand glutamine-rich (SFPQ) was not affected by arsenite-induced cellular stress, arsenite treatment induced the formation of cytoplasmic Rbfox2 granules in HeLa cells (arrowhead, Fig. 1). In order to determine the composition of these Rbfox2-containing cytoplasmic granules, Rbfox2 was co-immuno-stained with two SG markers, TIA-1 and G3BP1 and found to co-localize (Fig. 2a,b). However, Rbfox2-containing cytoplasmic granules were distinguished from GW182 (a 182 kDa protein with multiple glycine/tryptophan repeats) positive processing bodies (P-bodies) which are involved in mRNA decay (Fig. 2c). In addition, light chain 3 A/B (LC3A/B)-stained autophagy was also clearly separate from Rbfox2-containing cytoplasmic granules (Fig. 2d). These results support that Rbfox2 is a component of SGs. We further asked whether Rbfox2 is a bona-fide component of SGs using the strategy of trapping mRNP into polysomes with cycloheximide, an inhibitor of translation elongation, which stabilizes polysomes. HeLa cells were cultured in the presence of various doses of arsenite with cycloheximide. Cycloheximide led to a strong reduction of Rbfox2-containing SGs in various doses of arsenite (Fig. 3). Since an important property of SGs is that their formation is antagonistic to polysome formation^35^, these results suggest that Rbfox2 is a bona-fide component of SGs in arsenite-treated cells. In addition, we observed localization of Rbfox2 in G3BP1 positive SGs in human head and neck carcinoma HN13 cells, following arsenite treatment (Suppl. S1).

**Figure 4:**
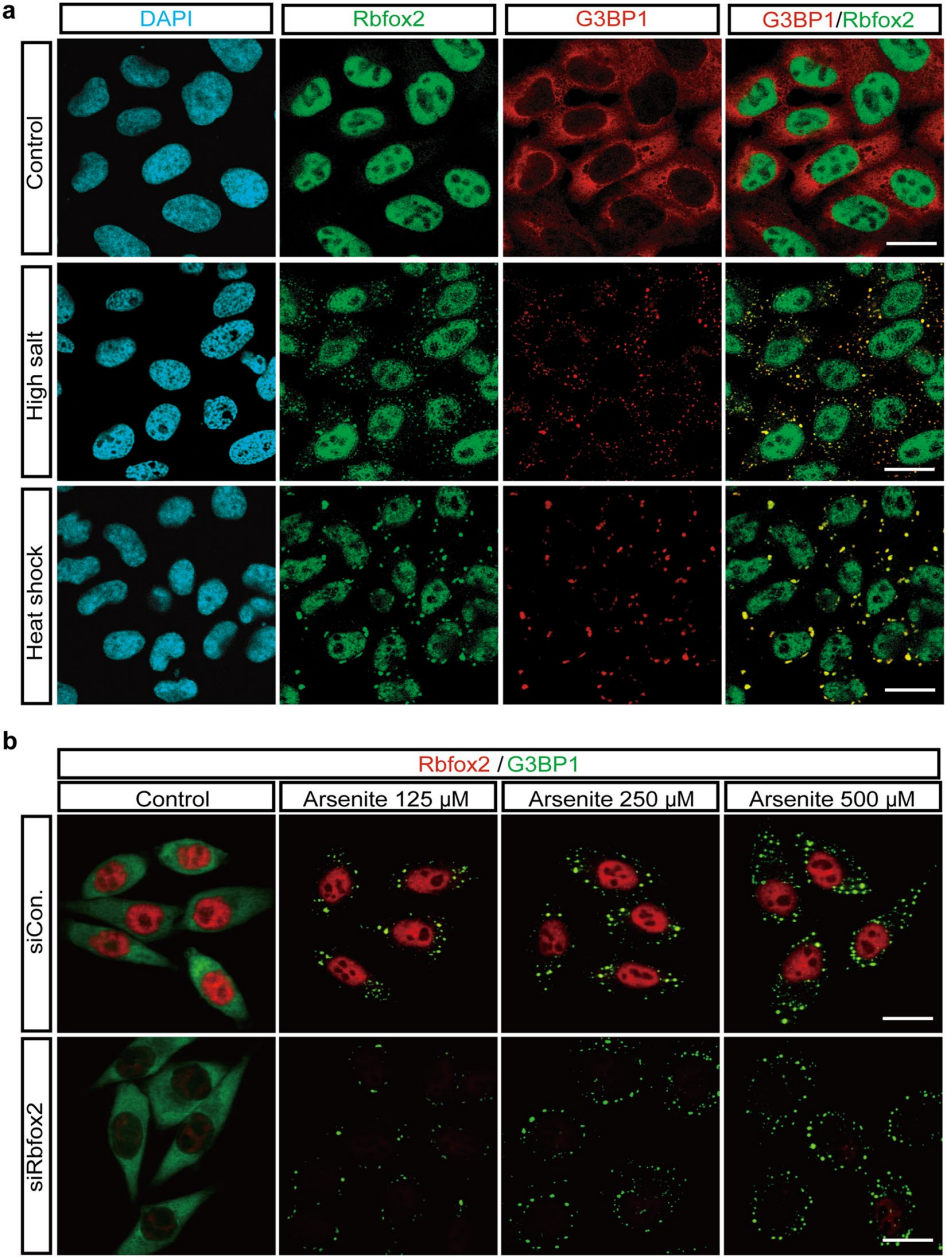
SG localization of Rbfox2 under various cellular stresses and effects of Rbdox2 depletion on SG formation. (**a**) Immunofluorescence images of Rbfox2 (green) and G3BP1 (red) proteins were visualized in indicated control and stress conditions in HeLa cells. (**b**) HeLa cells transfected with control siRNA (siCon.) or siRbfox2 for 36 hr were treated with indicated sodium arsenite concentrations for 40 min, and then immunofluorescence images of Rbfox2 (red) and G3BP1 (green) proteins were visualized. DAPI stains nuclei. Scale bar, 20 μm.

We then tested whether Rbfox2 localization into SGs could be induced by other cellular stresses. High salt and heat shock induced Rbfox2 to localize into G3BP1 positive SGs (Fig. 4a). We next asked whether Rbfox2 plays a role in SG formation. Depletion of Rbfox2 using a specific siRNA against endogenous Rbfox2 mRNA (siRbfox2) has no effect on SG formation in the presence of various doses of arsenite (Fig. 4b), indicating that Rbfox2 is not essential for SG formation. Next we examined the time course of SG assembly and disassembly in HeLa cells during exposure to and removal of cellular stress. Following 40 min of exposure to arsenite, cells were washed and allowed to recover in complete growth medium for various times. SGs were no longer detectable after a 3 h recovery period (Suppl. S2), showing that SG formation is highly dynamic and fully reversible upon removal of the stressor.

### Rbfox family proteins are recruited into SGs

**Figure 5:**
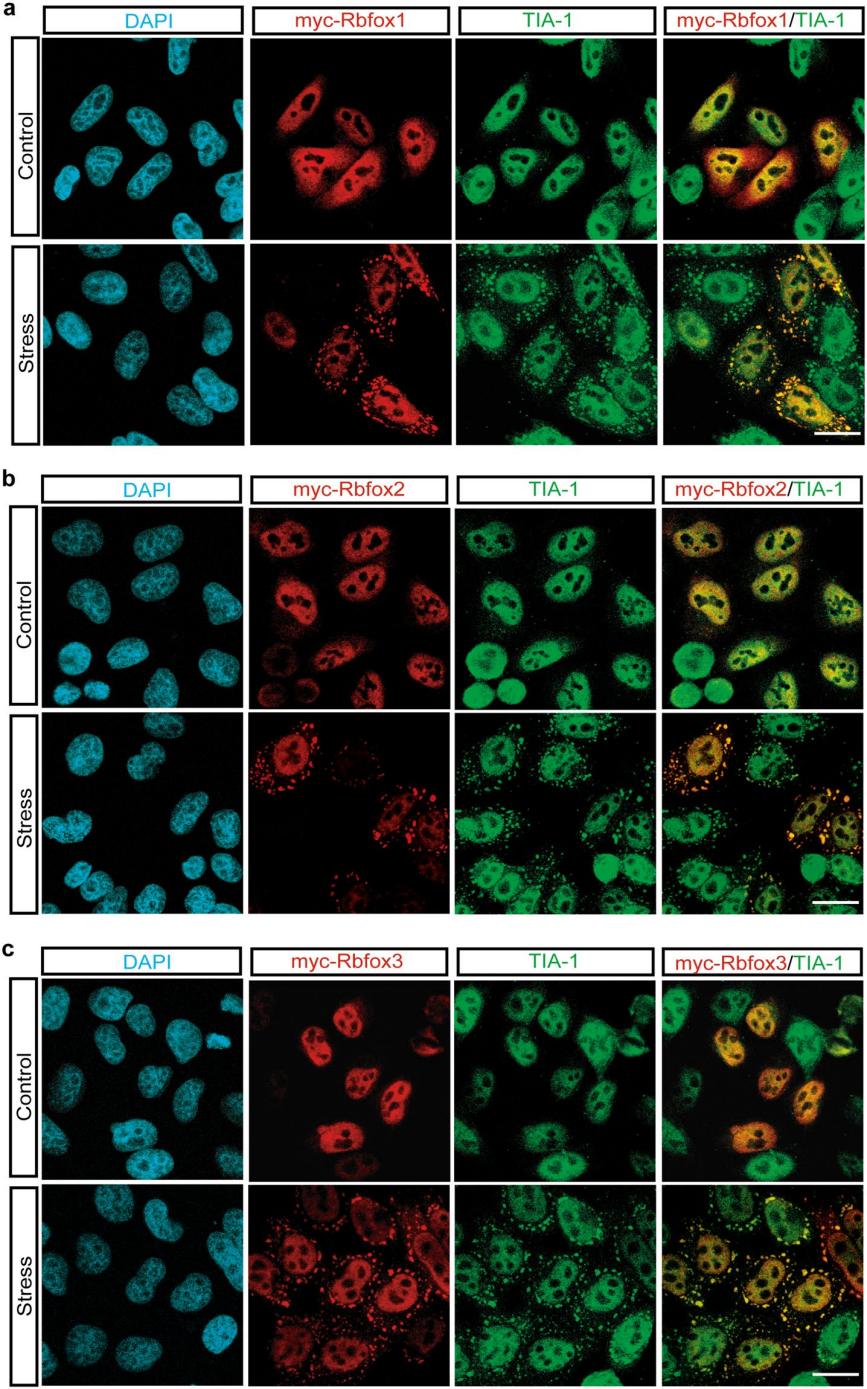
SG localization is a general property of Rbfox family proteins. (**a**–**c**) HeLa cells were transfected with constructs of myc-tagged Rbfox1, 2 and 3. Immunofluorescence images of myc-Rbfox (anti-myc, red) and TIA-1 (green) proteins were visualized in untreated (Control) and sodium arsenite-treated HeLa cells (Stress). DAPI stains nuclei. Scale bar, 20 μm.

Mammalian genomes encode three Rbfox members: Rbfox1, Rbfox2, and Rbfox3. All three proteins contain an almost identical RRM in the middle of the molecule. Although there are a number of alternatively spliced isoforms for each gene, the C-terminal domains of the major isoforms from the three genes also show a high sequence similarity. The high degree of sequence identity raised the possibility that Rbfox family proteins other than Rbfox2 may also localize into SGs. To address this possibility, expression plasmids of myc-tagged Rbfox1, Rbfox2, and Rbfox3 were transfected into HeLa cells, and the exogenously expressed proteins were monitored by immunofluorescence microscopy using anti-myc and the SG marker TIA-1 antibodies. Anti-Rbfox2 and anti-myc immunoblot results indicate the expression levels of endogenous and exogenous Rbfox2 proteins, respectively (Supple. S3). As previously reported^22, 36^, all three members of the Rbfox family showed strong nuclear localization in the absence of stress (Fig. 5a–c, upper rows, Control). Cellular stress induced SG localization of all exogenously expressed Rbfox family proteins (Fig. 5a–c, lower rows, Stress), suggesting that SG localization is a general property of Rbfox family proteins.

**Figure 6:**
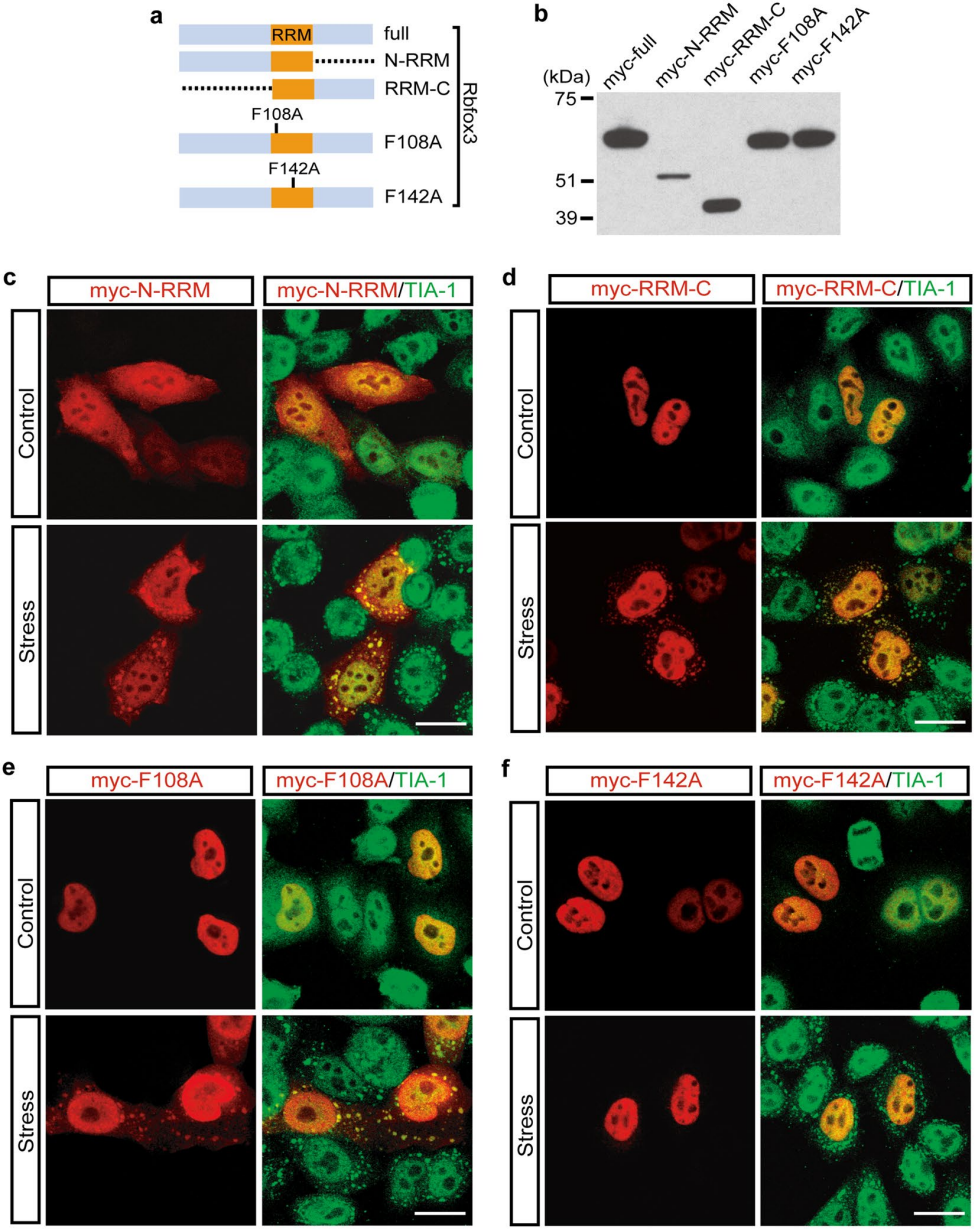
RNA binding activity of Rbfox family protein is crucial for its SG localization. (**a**) Diagram of myc-tagged deletion or mutation constructs of Rbfox3. (**b**) HeLa cells were transfected with indicated Rbfox3 constructs for 24 hr. Representative immunoblot with anti-myc showing the protein products from the expression plasmids. (**c**–**f**) HeLa cells were transfected with constructs shown in Fig. 6a. Immunofluorescence images of myc-Rbfox (anti-myc, red) and TIA-1 (green) proteins were visualized in untreated (Control) and sodium arsenite-treated HeLa cells (Stress). DAPI stains nuclei. Scale bar, 20 μm.

We next determined the domain that is responsible for the localization of Rbfox into SGs. Since the domains of the Rbfox3 protein had previously been investigated by us^6^, we decided to determine whether the N-terminal and C-terminal domains flanking the RRM are required for SG localization using Rbfox3 as representative of the Rbfox family. The N-terminal half of Rbfox3 including the RRM (N-RRM) and the C-terminal half (RRM-C) tagged with myc were expressed at their expected size, as shown in Fig. 6a and b. Although N-RRM, where the C-terminal domain was deleted, distributed diffusely in both nucleus and cytoplasm, it still localized into SGs upon arsenite treatment (Fig. 6c). Similarly, RRM-C, where the N-terminal domain was deleted, showed a strong nuclear localization property and localized into SGs (Fig. 6d). These results suggest that neither the N-terminal nor C-terminal domain of Rbfox3 is critical for SG localization. Next, we investigated whether the RNA binding activity of Rbfox3 through its RRM is required for SG localization using two different Rbfox3 mutant proteins. Rbfox family proteins contain two critical phenylalanine (F) residues within the RRM domains (amino-acids 108 and 142 in Rbfox3) that are required for RNA-binding activities. The F142 mutation to alanine (F142A) in Rbfox3 abrogates its binding activity to both the canonical binding motif UGCAUG and noncanonical target RNAs. The F108A Rbfox3 mutant protein is unable to bind canonical RNAs but is able to bind to noncanonical RNA targets^22^. Surprisingly, the F142A mutant protein completely lost the ability to localize into SGs (Fig. 6f), while the F108A mutant protein still localized into SGs (Fig. 6e). These results suggest that the RNA binding activity of the Rbfox family protein is crucial for its localization into SG and that Rbfox target RNAs recruited to SGs do not necessarily contain the canonical sequence.

### Rbfox2 binds to the mRNAs in SGs that are involved in the cell cycle

The fact that Rbfox is a component of SGs and its RNA binding activity is critical for its SG localization prompted us to investigate its role in SGs by identifying its target RNAs. To identify the RNA targets associated with the Rbfox2 protein, we performed an RIP-seq analysis using cytosolic fractions from arsenite-treated HeLa cells. Consistent with our observations that arsenite treatment induces Rbfox2 localization into cytosolic SGs, Rbfox2 proteins in the cytosolic fraction were increased by arsenite treatment (Suppl. S4). Moreover, the RNA-binding protein SFPQ, in agreement with the microscopy results shown in Fig. 1, was undetectable in the cytosolic fraction in both unstressed and stressed conditions, confirming the absence of nuclear contamination. Immunoprecipitation was carried out using either anti-Rbfox2 (“Bethyl” Laboratories, hereafter referred to as RIP-seq-A) or anti-Rbfox2 (produced in this laboratory “LMC”, hereafter referred to as RIP-seq-B) antibodies to exclude potential non-specific interactions and artifacts. Immunoprecipitated RNA (200–1,200 nt) was reverse transcribed to cDNA, ligated with adapters, PCR-amplified, and sequenced using an Illumina HiSeq.

**Figure 7:**
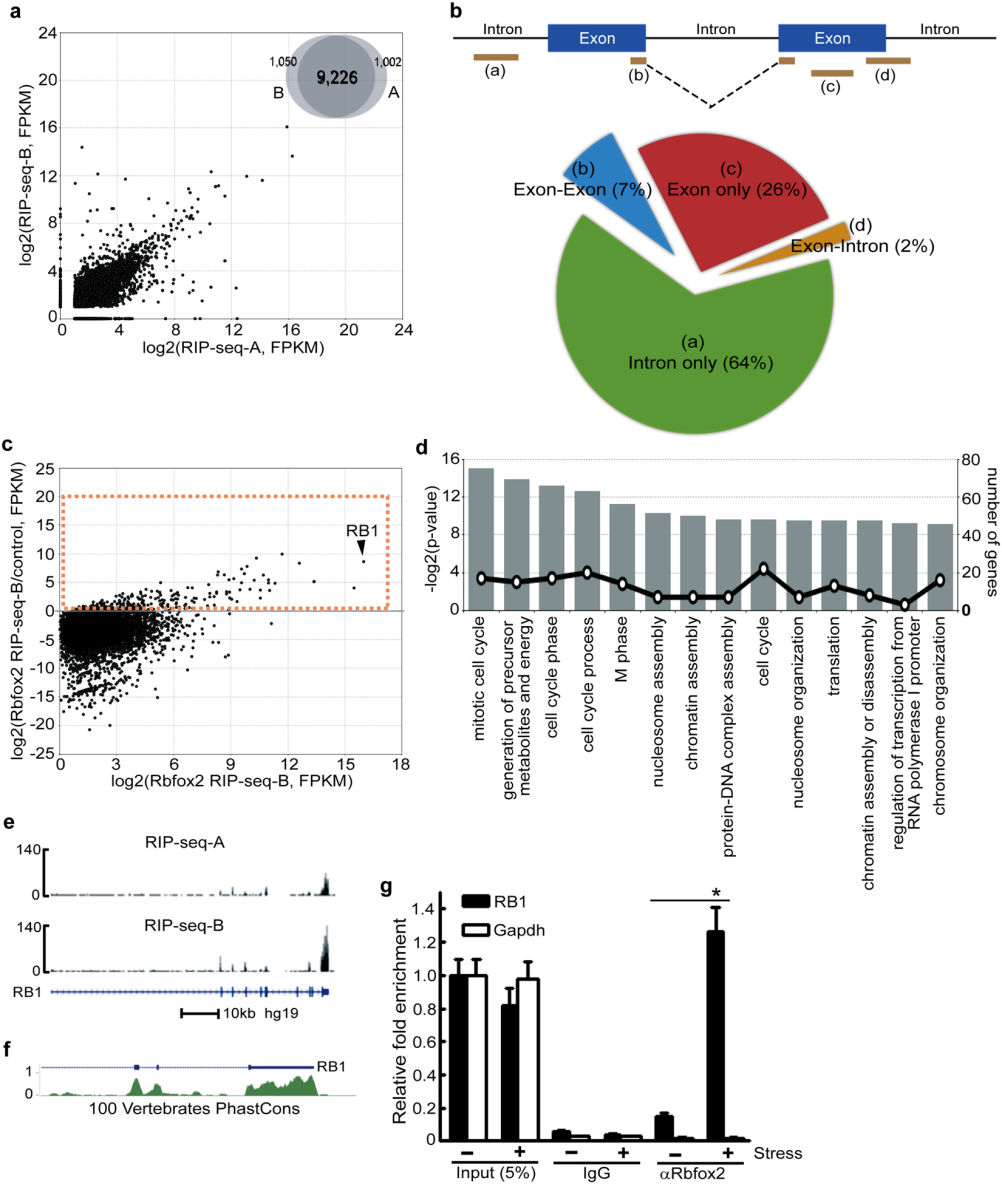
Identification of Rbfox2 target RNAs using RIP-seq analysis. (**a**) Correlation of RNA levels (log_2_(*FPKM*_*RIP*-*seq*_)) between two replicates. Venn diagram indicates the total number of Rbfox2 binding RNAs by both experimental conditions (RIP-seq-A and RIP-seq-B). (**b**) The distribution of Rbfox2 binding sites. (**c**) Correlation of expression level log fold change between Rbfox2 RIP-seq and control RNA-sequencing. The dashed rectangle indicates enriched RNAs (fold change ≥ 2) in Rbfox2 RIP-seq compared to control. (**d**) Gene Ontology (GO) analysis of enriched RNAs shown in the dashed rectangle of (**c**). The 14 most enriched GO biological process categories are shown. (**e**) An integrative genomics viewer (IGV) browser snapshot of Rbfox2 occupancy within the RB1 locus in two biological replicates (RIP-seq-A and RIP-seq-B) for RIP-seq analysis. (**f**) Phylogenetic conservation of 3′ UTR of RB1. Conservation of the RB1 targeting 3′ UTR is shown by the phastCons track on the UCSC Genome Browser in the human hg19 genome. The phastCons score estimates the probability of each nucleotide, using a hidden markov model-based method, belonging to conserved elements. This track shows multiple alignments of 100 vertebrate species and measurements of evolution conservation. (**g**) After treatment of HeLa cells with sodium arsenite, RIP-qRT-PCR (IP followed by qRT-PCR) analysis was used to measure the levels of enrichment of RB1 mRNA and GAPDH mRNA associated with immune-complexes using anti-Rbfox2 (αRbfox2) or control IgG; the data represented as enrichment of each mRNA in immuno-complexes were compared with 5% input. Mean values ± SD (error bars) are shown for three independent experiments. *P < 0.01 (two-sided *t* test), comparing stressed with unstressed RB1 mRNAs.

We obtained 23 and 28 million 50-nucleotide paired-end reads uniquely mapped to the human genome from RIP-seq-A and RIP-seq-B in arsenite treated cells, respectively (Supplementary Table S1). Two replicates of untreated HeLa cell total RNA used for controls generated 61 and 75 million reads uniquely mapped to the human genome (Supplementary Table S1). More than 90% of the target genes were identified in both RIP-seq data sets (Fig. 7a, Venn diagram), demonstrating the high reproducibility of the RIP-seq experiments. Thus, further analyses were performed on the data set of RIP-seq-B. Twenty eight and 72% of the reads were mapped to intergenic and genic regions, respectively. The binding regions of Rbfox2 at the genic regions are distributed as follows: 64% intron only, 26% exon only, 7% exon-exon junction and 2% exon-intron junction regions (Fig. 7b). Unexpectedly, we observed a large amount of intronic read hits, suggesting that incompletely spliced pre-mRNAs were released from the nucleus to the cytoplasm upon stress exposure. Although another possible explanation is that this observation is partly due to the presence of nuclear fractions, the presence of only 2% reads from exon-intron junction regions compared with 7% reads from exon-exon junction regions suggests that pre-mRNA contamination from nuclear fractions is not the main reason. To explicitly identify the transcripts of Rbfox2-binding targets, we made a conservative estimate of the enrichment of RNAs in RIP versus controls. To visualize the enrichment data, scatter plots were constructed using the log-transformed and normalized read numbers (Fig. 7c). The dashed rectangle represents the enriched genes in Rbfox2-RIP (Supplementary Table S2). A gene ontology (GO) analysis of the enriched genes identified multiple GO terms, of which the highly significant terms were related to the cell cycle (Fig. 7d). Among the enriched genes, retinoblastoma 1 (RB1) mRNA was identified as the most significant target of Rbfox2 (Fig. 7c). Moreover, we observed the enrichment of Rbfox2 binding to the 3′-UTR of RB1 mRNA in both RIP-seq analyses (Fig. 7e), and the sequence of the 3′-UTR of RB1 mRNA is highly conserved in vertebrates (Fig. 7f). All other hits with smaller numbers corresponded to exon regions but not to introns, suggesting that Rbfox2 associated with mature RB1 mRNA. Although the results obtained with the RRM-mutant Rbfox3 (Fig. 6e,f) suggest that Rbfox target RNAs do not necessarily contain the Rbfox consensus sequence in order to be recruited to SGs, the human 3′-UTR of RB1 mRNA contains two (U)GCAUG elements, one of which is conserved in mouse. We then confirmed the increase in binding between Rbfox2 and RB1 mRNA during oxidative stress using RIP-PCR analysis. After co-immunoprecipitation of Rbfox2-RNA complexes from HeLa cell extracts with or without arsenite-treatment, the 3′-UTR region of RB1 mRNA was amplified by RT-PCR. Rbfox2 binding to RB1 mRNA was greatly increased by arsenite treatment (Fig. 7g). This increment was not detected in the control IgG or Gapdh mRNA, confirming that Rbfox2 binds specifically to RB1 mRNA and that their interaction is increased by cellular stress.

### Rbfox2 regulates RB1 expression during and following stress exposure

**Figure 8:**
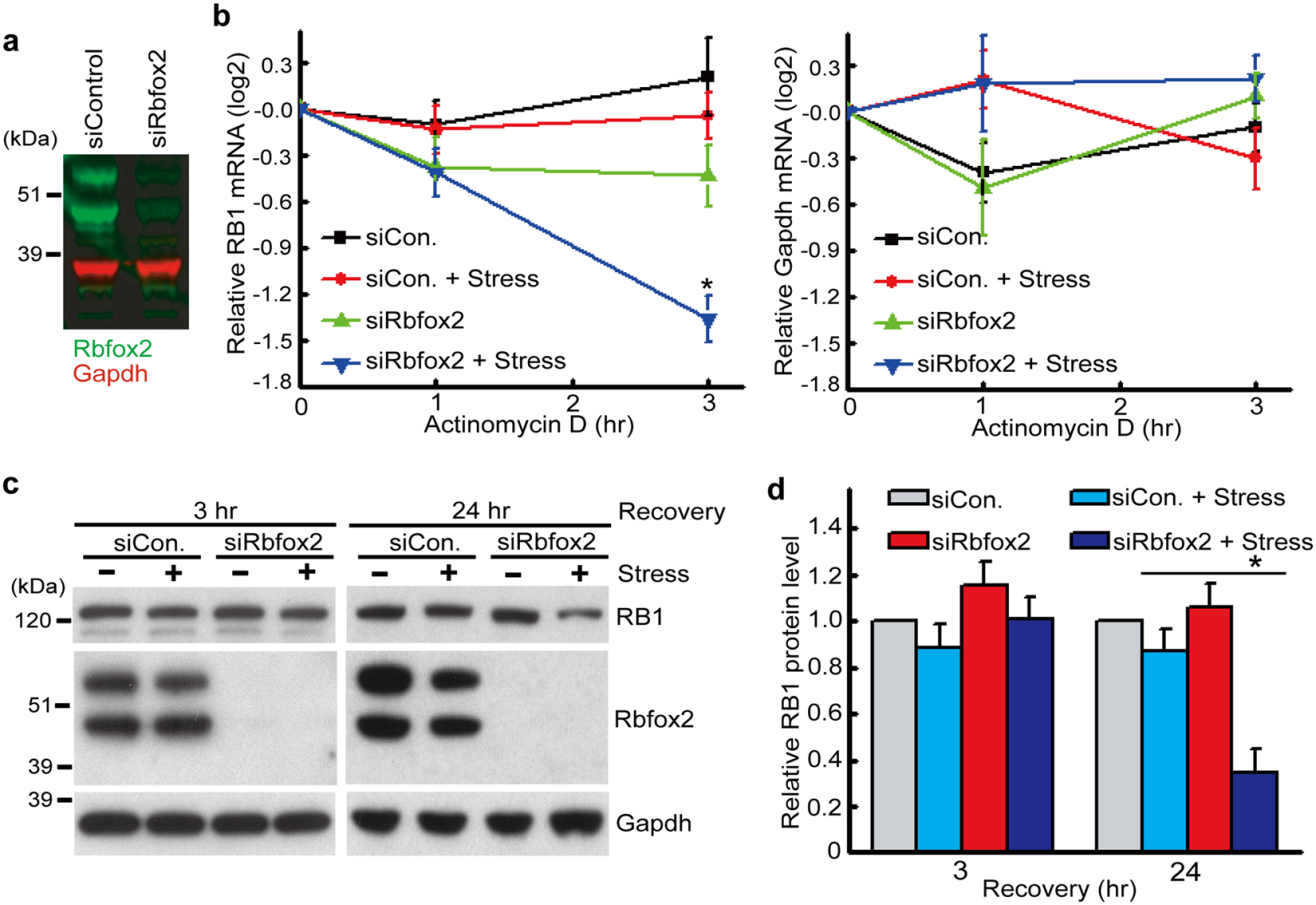
Rbfox2 affects RB1 protein expression under cellular stress. (**a**) HeLa cells were transfected with siCon. or siRbfox2 for 36 hr. Representative immunoblot showing the effects of siRNA transfections on protein levels of Rbfox2 (green) and Gapdh (red). (**b**) The steady- state levels of RB1 and GAPDH mRNAs were measured by qRT-PCR using total RNA from control HeLa cells (siCon.) and Rbfox2-deficient HeLa cells (siRbfox2) treated for 40 min with or without sodium arsenite (500 μM), followed by treatment for 0–3 hr with actinomycin D (2.5 μg/ml) in arsenite-free media. *P < 0.05, comparing stressed siRbfox2 with siCon. samples. (**c**,**d**) HeLa cells transfected with siCon. or siRbfox2 for 36 hr were treated with sodium arsenite, followed by recovery for 3 and 24 hrs. Cell lysates were subjected to immunoblot analysis using the indicated antibodies. Bands were quantified via densitometry and normalized to Gapdh. Mean values ± SD (error bars) are shown for three independent experiments. *P < 0.05 (two-sided *t* test), comparing stressed siRbfox2 with siCon. samples.

SGs function to protect selected mRNAs and suppress their translation under stress conditions and SGs dissociate when the stress is removed or when cells adapt to stress, thereby allowing SGs to play a role in protein expression during and following cellular stress. Therefore we investigated the role of Rbfox2 in RB1 expression by examining its ability to regulate RB1 mRNA stability and translation, using an siRNA strategy. Immunoblot analysis showed that endogenous Rbfox2 protein is efficiently depleted by the specific siRNA against Rbfox2 (Fig. 8a). No differences were observed in the basal expression level of RB1 mRNA upon Rbfox2 depletion (data not shown). We then measured the level of RB1 mRNA by quantifying the rate of decay after transcription was blocked by actinomycin D treatment. The level of RB1 mRNA was not altered by arsenite treatment in control cells. In contrast arsenite treatment significantly reduced the level of RB1 mRNA in Rbfox2-depleted cells by the 3 h recovery time, when SGs dissociated (Fig. 8b, left panel). On the other hand, the stability of a control stable transcript, Gapdh mRNA, encoding a housekeeping gene product, was not affected by Rbfox2 depletion and arsenite treatment (Fig. 8b, right panel), demonstrating that not all mRNA levels decreased rapidly following Rbfox2 depletion in stressed cells. These results suggest that Rbfox2 is required to protect RB1 mRNA from degradation presumably by incorporating RB1 mRNA into SGs under stress conditions. To further assess whether the binding of Rbfox2 to RB1 mRNA influences the protein level of RB1, immunoblot analysis, at two different recovery time points using anti-RB1 antibody, was carried out and the level of RB1 protein was determined. Although RB1 protein levels were the same in all conditions by 3 h of recovery, we observed reduced RB1 protein levels after Rbfox2 depletion and arsenite treatment at the 24 h recovery time point (Fig. 8c,d), presumably due to the reduced RB1 mRNA level. Our results demonstrate that Rbfox2 prevents RB1 mRNA degradation in SGs under cellular stress and that Rbfox2 is required to maintain RB1 protein expression in the post-stressed condition.

## Discussion

The goal of our study was to investigate the cytoplasmic function of Rbfox2 under stress conditions. Here, we used RIP-seq analysis to identify target RNAs of Rbfox2 in cytoplasmic SGs and found that genes affecting the cell cycle are significantly enriched in Rbfox2 targets. Our findings underscore the importance of a novel function of Rbfox2 that allows significant gene regulatory flexibility which, in turn enables cells to adjust to external conditions.

Each member of the Rbfox family of genes is known to generate a number of isoforms through splicing of multiple alternative exons and alternative promoters^36^. Both the N-terminal and C-terminal amino acid sequences affect their subcellular localization. Major isoforms from each gene predominantly localize in nuclei. Some isoforms with a reading frame shift at the C-terminus localize predominantly in the cytoplasm. However, to what extent each isoform of the Rbfox proteins shuttles between nucleus and cytoplasm has not been studied. In this report, we show that endogenous Rbfox2 isoforms and an exogenously expressed Rbfox2 isoform which are predominantly localized in nuclei are recruited to SGs in the cytoplasm under stress conditions. These observations suggest the idea that at least some isoforms of Rbfox2 translocate from the nucleus to the cytoplasm, although the amounts of translocated proteins by stress are small relative to the total amounts. Cytoplasmic form(s) of Rbfox2, which seems to be diffusely distributed in unstressed cells, might also be recruited to SGs, since the N-RRM deletion mutant of Rbfox3, which distributes to the cytoplasm to a significant extent, is also recruited to SGs. Although we have not formally tested Rbfox2 deletion mutants, it is likely that the N- and C-terminal domains of Rbfox2 are not essential for targeting to SGs, based on the data from Rbfox3 deletion mutants. It is also unlikely that a unique amino acid sequence of Rbfox2 is required for SG localization, because all exogenously expressed Rbfox1, 2, and 3 show recruitment into SGs to a similar extent. However, we cannot completely rule out the possibility that the Rbfox3 deletion mutant or full-length Rbfox3 and 1 forms a dimer (or multimer) with endogenous intact Rbfox2 through the RRM domain and is recruited to SGs together with endogenous Rbfox2. Of note, RNA binding activity is essential for Rbfox recruitment to SGs, whereas neither the N-terminal nor C-terminal domain is essential. This suggests that translocation of nuclear Rbfox to cytoplasmic SGs might be navigated by its target RNAs. This observation is in contrast to the fact that the C-terminal and/or N-terminal domains, in addition to the RNA binding activity, are required for regulation of alternative splicing in many cases^5, 6^. However, understanding the detailed mechanism by which Rbfox undergoes SG localization will require precise kinetic probing of Rbfox binding to target RNAs and determination of the molecular mechanism underlying posttranslational modification of Rbfox during cellular stress. To date cytoplasmic Rbfox1 has been reported to increase mRNA stability and translation by binding to the 3′-UTRs of its target RNAs in unstressed conditions, in some cases by competing with miRNAs^20^. Our direct evidence for SG localization of Rbfox2 during cellular stress provides a new gene regulatory mechanism for Rbfox.

In this study we provide comprehensive analysis of cytoplasmic Rbfox2-associated RNAs that are generated under oxidative stress. The fact that more than half of genic reads are mapped to introns suggests a leak of incompletely processed pre-mRNAs from the nucleus and/or a splicing inhibition under the stress condition. It is also possible that the number of intron-mapped reads is apparently large because intron length is generally much longer than exon length. Shalgi *et al*. reported widespread inhibition of pre-mRNA splicing during heat stress and nuclear retention of intron-containing transcripts^37^. Interestingly, they observed splicing-inhibited transcripts were mostly spliced posttranscriptionally in the nucleoplasm and large intron-containing transcripts are more susceptible to splicing inhibition. The physiological significance of intron-containing RNAs associated with cytoplasmic Rbfox2 will be determined in a future work.

Importantly, we found that the target RNAs of cytoplasmic Rbfox2 are related to the cell cycle progression. Among Rbfox2-target RNAs in SGs, we demonstrated the significance of RB1 mRNA association with Rbfox2 to regulate RB1 mRNA stability and RB1 protein expression under oxidative stress. The generally accepted roles of SGs are to provide a pause in translation of incorporated mRNAs and to protect them from degradation^25, 26^. SGs are transient and reversible structures. Upon removal of stress or adaptation of cells in a new environment, SGs are disassembled and then the protected mRNAs become readily available for translation. RB1 was initially identified as a tumor suppressor gene that is dysfunctional in several major cancers^38^. Since abnormal cells, including cancer cells, are under various cellular stresses such as oxidative stress, nutrient stress, and osmotic stress, we speculate that regulation of the RB1-mediated cell cycle by Rbfox2 could play a role in disease development and cancer progression.

Cells are constantly encountering numerous stresses such as an excess in hormone secretion, viral infections, oxidative stress, exposure to an abnormal pH, heat shock, hypoxia, and high salt concentrations. Recently, it was reported that cytoplasmic Rbfox3 levels were significantly higher in brain sections obtained from patients infected with human immunodeficiency virus type I (HIV) compared to those obtained from controls^39^. Although SG localization of HIV-induced-cytoplasmic Rbfox3 has not been confirmed, this is consistent with our observation that the subcellular localization of Rbfox2 is altered during cellular stress. Since the Rbfox family proteins are highly expressed in neuronal cells and all three members of this family are capable of responding to cellular stress, Rbfox proteins might play a role in development or prevention of neurodegenerative diseases which are often triggered by cellular stresses.

## Materials and Methods

### Cell culture, transfection, and chemicals

The human cervix adenocarcinoma cell line HeLa was maintained in Dulbecco’s modified Eagle medium (DMEM) supplemented with 10% fetal bovine serum and antibiotics. Cells were transfected with plasmids or siRNA by electroporation (Amaxa Nucleofector, Lonza). Transfected cells were generally analyzed 36 hr later. Sodium arsenite and sodium chloride were purchased from Sigma-Aldrich. Stress conditions were induced by treating cells with different stress inducers such as 500 μM sodium arsenite, 150 mM sodium chloride, or 43 °C heat shock for 40 min.

### Plasmids and siRNA

The preparation of the expression plasmids for Rbfox1, Rbfox2, and Rbfox3 in the pCS3 + MT vector, which contains an N-terminal myc-tag, has been described previously^7, 36^. The N-RRM and RRM-C deletion constructs of Rbfox3 have been described previously^6^. The generation of F108A and F142A mutant clones of Rbfox3 has been described previously^22^. The generation of the siRNA designed for silencing the Rbfox2 (siRbfox2) has been described previously as a Fox2 siRNA^6^.

### RNA-protein complex immunoprecipitation sequencing (RIP-seq) and PCR

A cytosolic fraction of sodium arsenite-treated HeLa cells was isolated for SG enrichment using a Nuclear Extract Kit (Active Motif). Immunoprecipitation of RNA-Rbfox2 complexes was performed with an RNA ChIP-IT kit (Active Motif) using two different Rbfox2 antibodies; one obtained from Bethyl Laboratory, A-300-864A, for RIP-seq-A and a second generated in this laboratory (LMC)^6^ for RIP-seq-B. The RNAs recovered from the complexes were subjected to cDNA-library preparation using a TruSeq Standard Total RNA library Prep Kit (Illumina). An Illumina Hiseq. 2000 platform was used to generate 100-bp and 50-bp paired-end reads for RIP-sequencing of Rbfox2 and control RNA-sequencing of total RNAs from untreated HeLa cells, respectively. For RIP-PCR, the RNAs recovered from the complexes of whole-cell extracts were subjected to RT-PCR using the primers 5′-CCA TGG AGA AGG CTG GGG-3′ and 5′-CAA AGT TGT CAT GGA TGA CC-3′ for Gapdh mRNA and 5′-GTT GGT CCT TCT CGG TCC TT-3′ and 5′-CAA AGC AGA AGG CAA CTT GA-3′ for RB1 mRNA.

### Bioinformatics analysis of RIP sequencing

After the sequencing run, the resulting raw reads in FASTQ format were processed using in-house Perl scripts. This enabled clean reads to be obtained by removing those containing adapter sequences, poly-N sequences, or low quality bases (below a mean Phred score of 20). The trimmed paired-end reads were mapped to the UCSC human reference genome (hg19) using Tophat 2^40^ under the default parameter setting. Only uniquely map-able reads with a maximum of two mismatches when compared with the reference were considered. The mapped results were fed to Cufflinks^41^ for quantification of known human genes. Differential expression analysis was performed with Cuffdiff^42^. Genes with a false discovery rate (FDR) of less than 5% were marked as significant. Unless otherwise stated, all gene expression levels in our analyses are represented as FPKM (fragments per kilobase of exon per million sequences fragments) values on log 2 scales. The RNA-sequencing statistics on all four samples are listed in Supplementary Table S1.

### Immunofluorescence microscopy

Cells were fixed with 4% paraformaldehyde for 10 min, permeabilized with 0.5% Triton X-100 in phosphate-buffered saline (PBS) for 15 min, blocked with 5% goat serum for 1 h and incubated overnight at 4 °C with primary antibodies recognizing G3BP1 (1:100, Santa Cruz Biotechnology, sc-365338), TIA-1 (1:100, Santa Cruz Biotechnology, sc-1751), SFPQ (1:1,000, Sigma P2860), GW182 (1:100, Santa Cruz Biotechnology, sc-56314), LC3A/B (1:100, Abcam, ab128025), myc (1:5,000, Invitrogen, 46-0603), and Rbfox2 (1:1,000, LMC antibody). Alexa-488- and Alexa-594-conjugated goat antibodies against mouse and rabbit IgG (1:500, Invitrogen, A11001, A11005, A11008, and A11012) were used as secondary antibodies. Nuclei were co-stained with 4,6-diamidino-2-phenylindole (DAPI, 5 μg/ml). Images were acquired with a Zeiss LSM 510 Meta confocal laser-scanning microscope.

### Immunoblot analysis

Whole cell lysates were prepared with M-PER buffer (Thermo Scientific) supplemented with a protease-inhibitor cocktail (Roche). Protein samples, denatured and reduced by sodium dodecyl sulfate (SDS) and β-mercaptoethanol respectively, were separated on SDS-polyacrylamide gels and transferred onto a nitrocellulose membrane. The SuperSignal system (Pierce) or Odyssey system (LI-COR) was used to detect the binding of secondary antibodies. Immunoblot bands were quantified using NIH ImageJ software. The primary antibodies recognized myc (1:5,000, Invitrogen, 46-0603), RB1 (1:500, Santa Cruz Biotechnology, sc-50), Rbfox2 (1:1,000, LMC antibody), and GAPDH (1:5,000, Biodesign, H86504M).

### Gene ontology (GO) analysis

We analyzed enrichment or depletion in Gene Ontology (GO) categories for the differentially expressed genes using the DAVID tool (https://david.ncifcrf.gov)^43^.

### Accession numbers

The RIP-sequence data have been deposited in the National Center for Biotechnology Information (NCBI) Sequence Read Archive under accession number SRP069155.

## Electronic supplementary material

Supplementary Information

Supplementary Table S1

Supplementary Table S2
