# Supplementary material for: Stress Granules Contain Rbfox2 with Cell Cycle-related mRNAs
Source: Sci Rep. 2017 Sep 11;7:11211. doi: 10.1038/s41598-017-11651-w (PMC5593835; doi:10.1038/s41598-017-11651-w)
Supplement: Supplementary file 1 — Supplementary Information [file 41598_2017_11651_MOESM1_ESM.pdf]

Stress Granules Contain Rbfox2 with Cell Cycle-related mRNAs

Chungoo Park[1], Sunkyung Choi[2], Yong-Eun Kim[2], Siyeo Lee[2], Su-Hyung Park[3], Robert S. Adelstein[4], Sachiyo Kawamoto[4,*], and Kee K. Kim[2,*]

[1]School of Biological Sciences and Technology, Chonnam National University, Gwangju, 61186, Republic of Korea. [2]Department of Biochemistry, Chungnam National University, Daejeon, 34134, Republic of Korea. [3]Graduate School of Medical Science and Engineering, KAIST, Daejeon, 34141, Republic of Korea. [4]Laboratory of Molecular Cardiology, National Heart, Lung, and Blood Institute, National Institutes of Health, Bethesda, MD 20892, USA

[*]To whom correspondence should be addressed to K.K.K.(email: kimkk@cnu.ac.kr) or S.K. (email: kawamots@nhlbi.nih.gov)

SUPPLEMENTARY MATERIALS AND METHODS

Cell culture

The human cervix adenocarcinoma cell line HeLa and the human head and neck squamous cell carcinoma cell line HN13 (1) were maintained in Dulbecco's modified Eagle medium (DMEM) supplemented with 10% fetal bovine serum and antibiotics. Stress conditions were induced by treating cells with 500 μM arsenite for 40 min. Control was vehicle.

SUPPLEMENTARY REFERENCE

1. Cardinali, M., Pietraszkiewicz, H., Ensley, J.F. and Robbins, K.C. (1995) Tyrosine phosphorylation as a marker for aberrantly regulated growth-promoting pathways in cell lines derived from head and neck malignancies. *Int J Cancer*, **61**, 98-103.

Supplementary Figure 1

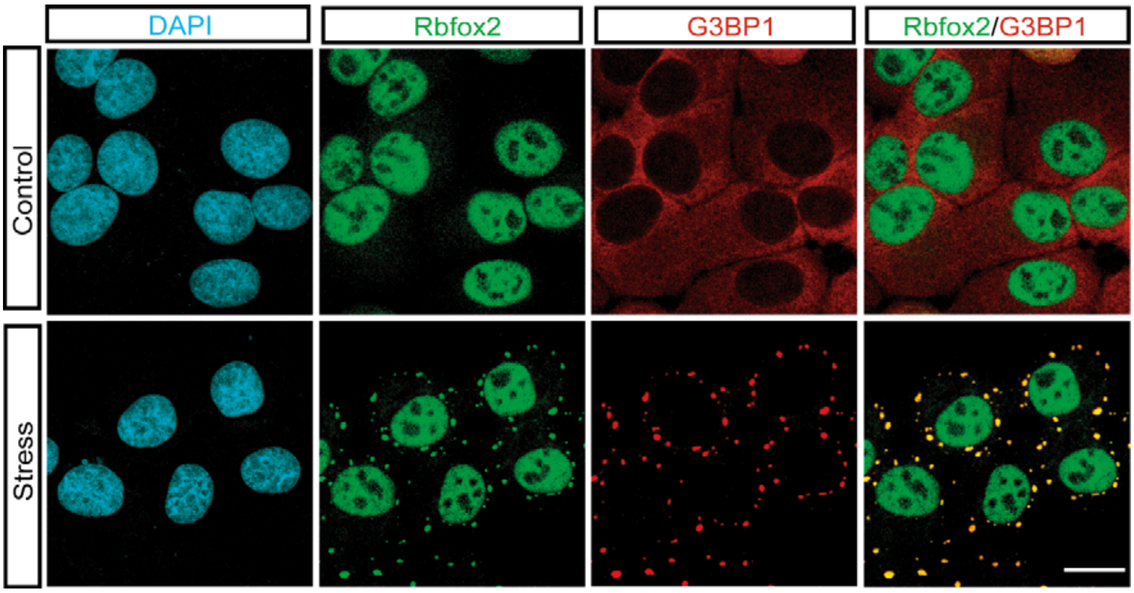

Figure S1. Recruitment of Rbfox2 into SGs in HN13 cells. Immunofluorescence images of Rbfox2 (green) and G3BP1 (red) proteins were visualized in untreated (Control) and sodium arsenite-treated (500 µM; 40 min) human head and neck carcinoma HN13 cells (Stress). DAPI stains nuclei. Scale bar, 20 µm.

Supplementary Figure 2

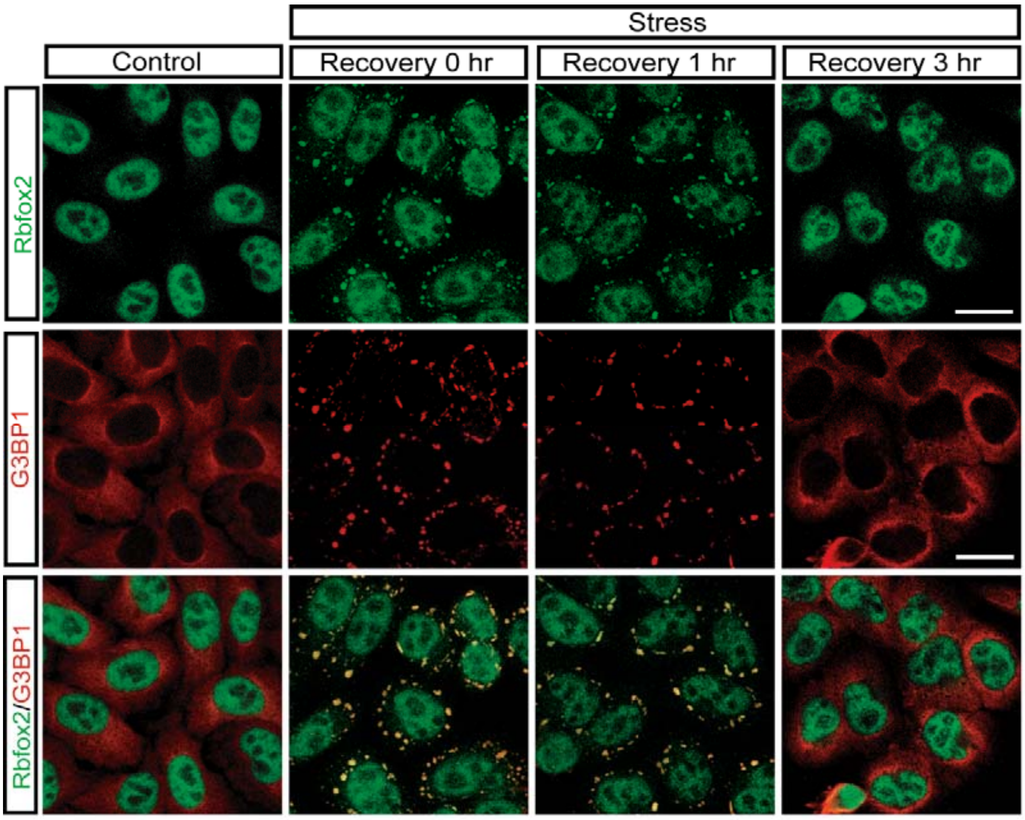

Figure S2. SG formation is dynamic and reversible. Sodium arsenite-treated HeLa cells were placed in complete media without sodium arsenite for the indicated time, then immunofluorescence images of Rbfox2 (green) and G3BP1 (red) proteins were visualized. Scale bar, 20 μm.

Supplementary Figure 3

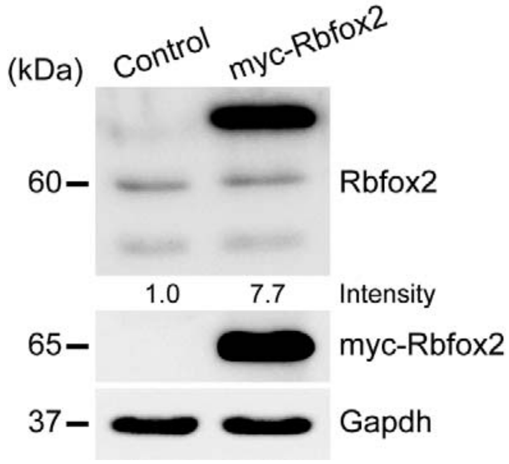

Figure S3. Endogenous and exogenous Rbfox2 protein levels. Total cell lysates from HeLa cells transfected with the empty vector (Control) and myc-Rbfox2 construct were subjected to immunoblot analysis using the indicated antibodies. Anti-Rbfox2 immunoblot indicates the expression levels of both endogenous and exogenous Rbfox2 proteins (upper panel). Anti-myc confirms exogenous myc-Rbfox2 (middle panel). Gapdh serves as a loading control (lower panel).

Supplementary Figure 4

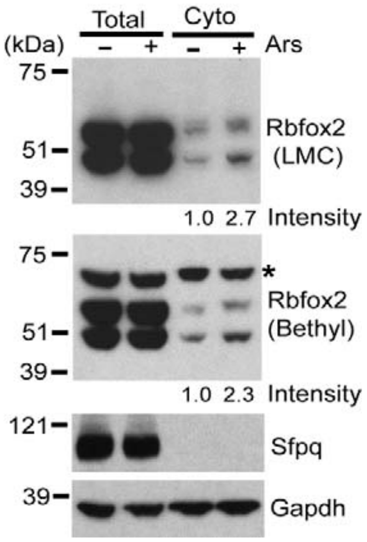

Figure S4. Validation of cytosolic fractionation and stress-induced increase in the cytosolic Rbfox2 level. Total cell lysates (Total) and cytosolic fractions (Cyto) from untreated and sodium arsenite-treated HeLa cells were subjected to immunoblot analysis using the indicated antibodies. *, non-specific signal.

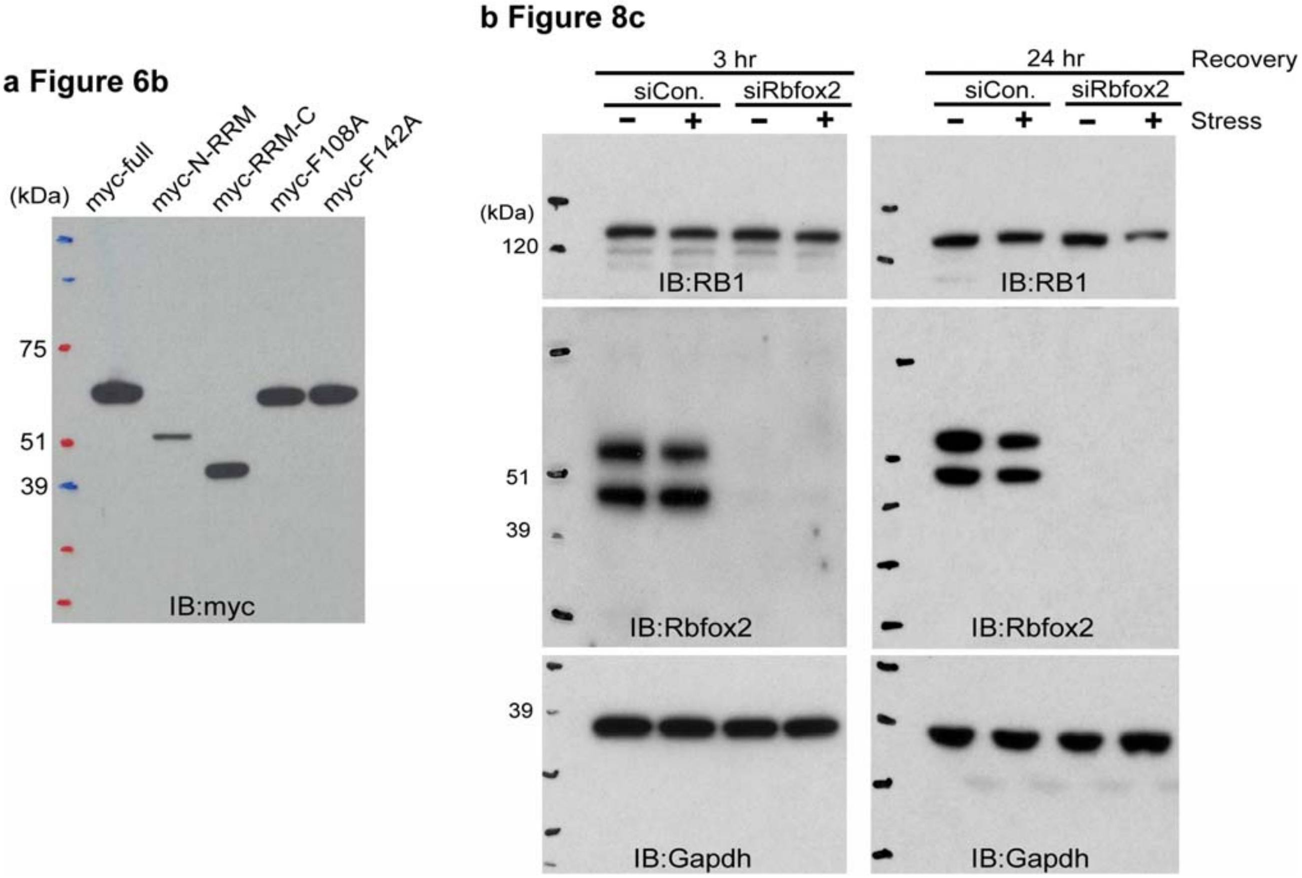

Figure S5. Uncropped images of immunoblots.

SUPPLEMENTARY TABLE LEGENDS

Supplementary Table S1. Mapping statistics of control RNA-sequencing and Rbfox2 RIP-sequencing

Supplementary Table S2 List of Rbfox2-binding mRNAs in the stress granules determined by RIP-sequencing.
